# Warfarin and fibrinolysis - a challenging combination: an observational cohort study

**DOI:** 10.1186/1757-7241-19-21

**Published:** 2011-04-05

**Authors:** Sini Saarinen, Jyrki Puolakka, James Boyd, Taneli Väyrynen, Harri Luurila, Markku Kuisma

**Affiliations:** 1 Helsinki Emergency Medical Service System, Helsinki University Central Hospital, PL 112, 00099 Helsinki City, Helsinki, Finland; 2 Helsinki EMS Research Group, Helsinki University Central Hospital, PL 112, 00099 Helsinki City, Helsinki, Finland; 3 Department of Cardiology, Helsinki University Central Hospital of Meilahti, 00290 Helsinki, Finland

## Abstract

**Background:**

Patients presenting with ST-segment elevation myocardial infarction (STEMI) frequently use warfarin. Fibrinolytic agents and warfarin both increase bleeding risk, but only a few studies have been published concerning the bleeding risk of warfarin-prescribed patients receiving fibrinolysis. The objective of this study was to define the prevalence for intracranial haemorrhage (ICH) or major bleeding in patients on warfarin treatment receiving pre-hospital fibrinolysis.

**Methods:**

This was an observational cohort study. Data for this retrospective case series were collected in Helsinki Emergency Medical Service catchment area from 1.1.1997 to 30.6.2010. All warfarin patients with suspected ST-segment elevation myocardial infarction (STEMI), who received pre-hospital fibrinolysis, were included. Bleeding complications were detected from Medical Records and classified as ICH, major or minor bleeding.

**Results:**

Thirty-six warfarin patients received fibrinolysis during the study period. Fourteen patients had bleeding complications. One (3%, 95% CI 0-15%) patient had ICH, six (17%, 95% CI 7-32%) had major and seven (19%, 95% CI 9-35%) had minor bleeding. The only fatal bleeding occurred in a patient with ICH. Patients' age, fibrinolytic agent used or aspirin use did not predispose to bleeding complications. High International Normalized Ratio (INR) seemed to predispose to bleedings with values over 3, but no statistically significant difference was found.

**Conclusions:**

Bleedings occur frequently in warfarin patients treated with fibrinolysis in the real world setting, but they are rarely fatal.

## Background

Pre-hospital fibrinolysis is an effective alternative in the treatment of acute ST-segment elevation myocardial infarction (STEMI). Reduced time delay from the onset of symptoms to fibrinolysis is related to reduced mortality [1,2]. Many patients presenting with STEMI also have other diseases, such as atrial fibrillation or severe heart failure, requiring oral anticoagulants for the prevention of thromboembolic complications. Guidelines of European Society of Cardiology, American College of Cardiology (ACC) and American Heart Association (AHA) consider the use of oral anticoagulants as a relative contraindication for fibrinolysis [3-5]. The most threatening complication associated with both warfarin and fibrinolytic agents is an intracranial or a major bleeding, which can be fatal. These patients are frequently transported directly to primary percutaneous coronary intervention (PCI). According to the guidelines, fibrinolysis should, however, be considered if PCI cannot be performed within 90-120 minutes when emergency medical service (EMS) meets the patient [3-5]. Additionally, the information on prior warfarin usage is not always available, especially in patients who are resuscitated from sudden out-of-hospital cardiac arrest.

Even though bleeding associated with fibrinolysis has been carefully investigated, only a few studies have been done concerning patients on oral anticoagulants receiving fibrinolysis. The aim of this study was to report the prevalence of serious haemorrhages with patients on oral anticoagulants receiving pre-hospital fibrinolysis for suspected STEMI.

## Methods

This was an observational cohort study approved by Institutional Review Board of Helsinki University Central Hospital. The study plan was retrospective, but data was collected prospectively for Fibrinolysis Registry in Helsinki EMS area during 1.1.1997-30.6.2010. Helsinki is the capital city of Finland with 584 000 inhabitants. The EMS consists of seven to eight basic life support (BLS) -units, four advanced life support (ALS) -units, a medical supervisor unit and a physician staffed mobile intensive care unit (MICU). The medical supervisor unit and the MICU are provided with fibrinolytic agents.

All patients on warfarin were included, if they received out-of-hospital fibrinolysis for suspected STEMI during the study period. Unlike many other studies concerning bleeding complications, this was a "real world setting" study including all patients despite their age, bleeding risk or clinical condition. The decision to initiate fibrinolysis for suspected STEMI was made by an emergency physician, either on scene or after consultation with 12-lead electrocardiogram (ECG) transmission. Emergency physicians in Helsinki EMS filled a detailed documentation form for Fibrinolysis Registry after each pre-hospital fibrinolysis. All patients receiving fibrinolysis for suspected STEMI or pulmonary embolism are included in the Fibrinolysis registry of Helsinki EMS, as well as patients with suspected STEMI receiving other treatment than fibrinolysis (i.e. primary PCI). Only patients receiving fibrinolysis for suspected STEMI were included in the study. STEMI diagnose was not confirmed from Hospital Medical Records. Therefore it is possible some patients received fibrinolysis inappropriately not actually suffering from STEMI. However, a physician responsible for maintaining the registry checked correctness of indications for fibrinolysis and ECG diagnostics in all cases.

One of the authors (J.P) investigated the Medical Records of receiving hospitals to detect possible complications after fibrinolysis. Reported laboratory tests were drawn on arrival to hospital. Bleeding complications were classified as intracranial haemorrhage (ICH), major and minor bleeding. ICH was diagnosed by computer tomography (CT) scan or as an autopsy finding. Major bleeding was defined as a haemorrhage causing a need for a blood-transfusion, all other bleedings reported in Medical Records were defined as minor. Time from fibrinolysis to bleeding, blood units transfused and treatment of bleeding were also registered.

Statistical analysis was performed using SPSS for Windows V18.0 Software (SPSS Inc, Chicago, IL, USA). Chi-Square test (Fisher's Exact Test) was used for categorical variables and Mann-Whitney U test for continuous variables with non-normal distribution. Median values were reported with 25th-75th percentiles and proportions with 95% confidence intervals (95% CI) according to the Agresti-Coull method. For statistical analysis ICH and major bleeding groups were compared to minor and no-bleeding groups. These two groups were chosen to be compared on the basis of clinical relevance.

## Results

### Study population

Altogether 1322 pre-hospital fibrinolysis for suspected STEMI were given during the study period. The study population consisted of 36 warfarin treated patients, whose baseline characteristics are shown in Table 1. Eighty three percent (95% CI 68-93%) of study patients were treated with fibrinolysis before year 2005. Chest pain was the most common presenting symptom (n = 24; 67%, 95% CI 50-80%). The main location of ST-segment elevation was anterior wall in 18 (50%, 95% CI 34-66%), inferior wall in 15 (42%, 95% CI 27-58%) and lateral wall in 3 (8%, 95% CI 2-23%) patients. Median time from the onset of the symptoms to fibrinolysis was 68 min (IQR 49-113 min), while median time from emergency call to fibrinolysis was 51 min (IQR 36-60 min). Other contraindications for fibrinolysis than warfarin existed with four (11%, 95% CI 4-26%) patients. Those were malignancy, hypertension and previous ICH.

**Table 1:** Baseline characteristics of study patients (n = 36)

n (%, 95% CI)	
Age (years)*	70 (62-78)
Sex; men	28 (78%, 62-89%)
Previous medical history	
ischaemic heart disease	21 (58%, 42-73%)
hypertension	24 (67%, 50-80%)
diabetes	11 (31%, 18-47%)
myocardial infarction	17 (47%, 32-63%)
thrombolysis	9 (25%, 14-41%)
PCI or CABG	5 (14%, 6-29%)
use of aspirin	2 (6%, 1-19%)
Signs before fibrinolysis	
cardiac arrest	10 (28%, 16-44%)
pulmonary oedema	1 (3%, 0-15%)
cardiogenic shock	5 (14%, 6-29%)
Warfarin	
prior use known before fibrinolysis	34 (94%, 81-99%)
indication	
atrial fibrillation	20 (55.5%, 40-70%)
cerebrovascular disease	6 (17%, 7-32%)
pulmonary embolism	1 (3%, 0-15%)
deep vein thrombosis	2 (5.5%, 1-19%)
heart valve disease	3 (8%, 2-23%)
cardiomyopathy	4 (11%, 4-26%)

* = median (IQR). PCI = Percutaneous Coronary Intervention, CABG = Coronary Artery Bypass Graft Surgery.

### Medication

Four fibrinolytic agents were used: streptokinase 1997-2002, alteplase (tPA) 1997, reteplase 1998-2007 and tenecteplase 2008-2010 (Table 2). As adjuvant medication, aspirin 250 mg and/or unfractionated (UFH) or low molecular weight heparin (LMWH) were used (Table 2). UHF was given 5000 IU as a bolus and continued with infusion 1000 IU/h. LMWH was enoxaparine, which was given 30 mg intravenously and 1 mg/kg subcutaneously or only subcutaneously or intravenously. UFH was replaced by LMWH after year 1999.

**Table 2:** Comparison between patients with major bleeding or ICH, minor bleeding and no bleeding

	ICH or major bleeding, n = 7	Minor bleeding, n = 7	No bleeding, n = 22
Age (years)*	69 (63-72)	67 (61-78)	70 (62-79)
Sex; men	7 (100%)	5 (70%)	16 (73%)
Earlier fibrinolysis	0	3 (43%)	6 (27%)
Contraindication for fibrinolysis (other than warfarin)	1 (14%)	0	3 (18%)
Systolic BP*	126 (105-141)	127 (90-140)	122 (111-140)
Diastolic BP*	64 (61-92)	69 (60-81)	76 (70-90)
Fibrinolytic agent			
streptokinase	1 (14.3%)	0	5 (23%)
tPA (alteplase)	1 (14.3%)	1 (14.3%)	1 (4.5%)
reteplase	5 (71.4%)	5 (71.4%)	13 (59%)
tenecteplase	0	1 (14.3%)	3 (13.5%)
Adjuvant medication			
aspirin	1 (14%)	3 (43%)	15 (68%)
heparin	5 (71%)	5 (71%)	14 (64%)
UFH	3 (43%)	1 (14%)	2 (9%)
LMWH	2 (29%)	4 (57%)	12 (55%)
LMWH (i.v.+s.c.)	1 (14%)	2 (29%)	3 (14%)
LMWH only i.v.	1 (14%)	2 (29%)	6 (27%)
LMWH reduced dose s.c	0	0	3 (14%)
Laboratory parameters at hospital arrival			
thrombocytes (x10^9^/l)*	168 (144-191)	205 (165-227)	161 (134-188)
haemoglobin (g/l)*	153 (133-163)	145 (139-155)	136 (125-141)
Survival			
hospital discharge	5 (71%)	5 (71%)	16 (73%)
30 days	5 (71%)	5 (71%)	14 (64%)
1 year	5 (71%)	4 (57%)	13 (59%)

* = median value (IQR), ICH = intracranial haemorrhage, BP = blood pressure, UFH = unfractionated heparin, LMWH = low molecular weight heparin, i.v. = intravenously, s.c. = subcutaneously.

### Laboratory test values

**Figure 1:**
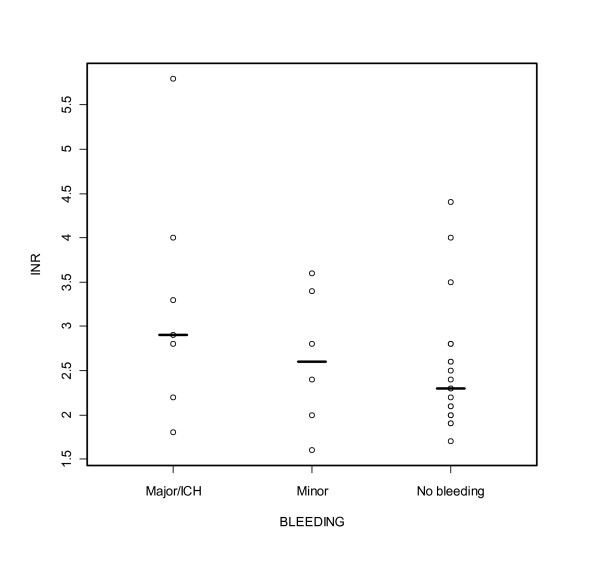
**Individual and median INR values with ICH/major, minor and no-bleeding groups**. Median INR for ICH/major bleeding group was 2,9 (IQR 2,5-3,7), for minor bleeding group 2,6 (IQR 1,9-3,5) and for no-bleeding group 2,3 (IQR 2,0-2,8).

On arrival to hospital, INR varied from 1.6 to 5.8. Median INR value was higher with ICH/major bleeding group compared to minor and no-bleeding groups (Figure 1). Median haemoglobin and thrombocyte count values are shown in Table 2.

### Bleeding complications

Bleeding complication occurred in 14 (39%, 95% CI 25-55%) patients, of whom only one had a bleeding before hospital admission. Median time from fibrinolysis to bleeding was 22 h (IQR 6-47 h). Major bleeding occurred in 6 (17%, 95% CI 7-32%) patients and ICH in one patient (3%, 95% CI 0-15%). The site of major bleeding was gastrointestinal in 3 (50% of major bleedings) patients, unknown in 2 and pharynx in one. In addition to gastrointestinal bleeding, haemothorax was diagnosed in one patient. Major-bleeding patients received on median 4 units of packed red blood cells. The sites of minor bleeding were puncture site, urinary or respiratory tract or ocular angle.

### Statistical findings

Statistically significant differences between ICH or major bleeding - and minor or no-bleeding groups on age, gender, blood pressure before fibrinolysis or heparin/LMWH use were not found. Warfarin patients who were medicated with aspirin prior to fibrinolysis, had less bleedings than those who did not receive aspirin (p = 0.037). Reteplase and tenecteplase use did not cause significantly less serious bleedings compared to other fibrinolytic agents (Table 2). A trend toward increasing bleeding rates with high INR existed: median INR was 2.9 with ICH or major bleeding patients, when no-bleeding patients' median INR was 2.3 (Figure 1). With INR-value 2-3, 18% (95% CI 5-42%) had a severe bleeding complication (ICH/major bleeding), whereas 38% (95% CI 13-70%) had a severe bleeding with INR >3. Statistical correlation between INR and bleedings was not found.

### Survival

Chest pain was relieved or ceased in 26 (72%, 95% CI 56-84%) patients before hospital admission and ST-segment elevation diminished over 50% 60-90 min after fibrinolysis with 25 (69%, 95% CI 53-82%) patients. Two (6%, 95% CI 1-19%) patients went into cardiac arrest on scene after fibrinolysis. One of them died before hospital admission. One bleeding complication, ICH, was fatal. Thirty-day mortality was almost equal to 1-year mortality (Table 2).

## Discussion

Bleedings occurred frequently after fibrinolysis with patients on warfarin. Major bleeding or ICH occurred in 7 (19%, 95% CI 9-35%) patients in our study population. Only one of the bleedings, i.e. ICH, was fatal.

Previously major bleeding - according to Thrombolysis in Myocardial Infarction (TIMI) criteria [6] or requiring blood transfusion - has been reported to occur in 2.3% to 8.3% of patients receiving fibrinolysis [7-9]. Gusto III study consisting of over 15 000 patients showed the prevalence of major bleeding with reteplase and alteplase to be 5.9% and 6.2%, respectively [10]. Compared to the rates of ICH with non-warfarin patients receiving fibrinolysis in other studies (0.2-2.6%) [8-15], anticoagulated patients had slightly higher probability for ICH (3%, 95% CI 0-15%) in our study. According to Helsinki Fibrinolysis Registry the prevalence of fatal ICH after pre-hospital fibrinolysis in patients with suspected STEMI not receiving warfarin was 1% (5/539, 95% CI 0-2%) between 1997-2002 (unpublished registry data). Prevalence of spontaneous ICH and major bleeding in warfarin patients has been reported to be 0.25% and 1.1% yearly in a previous study [16].

Only a few studies concerning the use of fibrinolytic agents in anticoagulated patients are available. Stanley et al did not find significant difference in serious complications between warfarin and non-warfarin patients bleeding complications after fibrinolysis, but there was a trend towards a greater prevalence of serious bleedings with anticoagulated patients [17]. Their anticoagulated fibrinolysis patients' rate of any bleeding (4%) was noteworthy lower than we found in our material, but their amount of ICH (4%) was the same as in our study population (3%) [17]. Stanley et al found that age, aspirin use and repeated fibrinolysis increases bleeding complications, but in our material no connection between increasing bleeding rates and age or aspirin use appeared. In our study patients receiving aspirin had significantly lower rate of bleedings, but this might be explained by a coincidence or some confounding factors since the size of population was relatively small. Brass and co-workers investigated patients over 65 years old and found that warfarin increased the risk of ICH only if INR was over 4 [15], while in our population bleedings seemed to increase already with INR-values over 3, but no statistically significant difference existed. Jaegere and co-workers (1992) suggested 3.7-fold higher probability of ICH with patients on anticoagulants [18].

Patients on warfarin suffered from multiple diseases in our unselected "real world setting" study population; ischaemic heart disease and previous myocardial infarctions were common with them, as well as cardiogenic shock or need for CPR. This may partially explain high 30-days and 1-year mortalities in warfarin patients. Bleeding caused only one death in this group and it does not therefore explain the high mortality.

Although fibrinolysis predisposes patients to bleeding complications, the reported reduction in mortality with pre-hospital fibrinolysis must be taken to consideration. Terkelsen et al reported STEMI patients` mortalities (15.4%; 23.3%; 28.1%; 30.8%) to increase as the delay from emergency dispatching center call to beginning of reperfusion therapy (fibrinolysis or PCI) lengthens (0-60 min, 61-120 min, 121-180 min, 181-360 min) [19]. This supports the acceptance of bleeding complication risk with warfarin patients if PCI can not be performed within the recommended time limit (120 min) [3-5]. In identifying the high-risk patients for bleeding, knowing the level of anticoagulation is valuable information. Some of the rapid point-of-care laboratory analysators used in ambulances have the capability to measure INR.

This study is limited by its retrospective nature, small study population, and a lack of a control group. The number of warfarin patients' fibrinolysis reduced notably after year 2004, when Helsinki University Central Hospital of Meilahti started organised PCI treatment for STEMI patients 24 hours daily. Primary PCI is a common therapeutic practice with warfarin patients. Therefore it is difficult to collect a large enough population of warfarin patients treated with fibrinolytic therapy to show significant differences in variables predisposing to bleedings.

Also, several fibrinolytic agents and adjuvants were used over time and therefore linking a certain medication regimen to increased bleeding risk is difficult considering the low number of patients. However, the use of a various medication regimen reflects the changes in clinical practise.

## Conclusions

We conclude that with warfarin patients receiving fibrinolysis, bleedings are common, but only a few of them are fatal. The level of anticoagulation is important knowledge before giving fibrinolysis, because bleedings seem to increase with high INR-values.

## Competing interests

The authors declare that they have no competing interests.

## Authors' contributions

SS has analysed the data and drafted the manuscript. JP has collected the data and has involved in revising the manuscript. JB and TV have given assistance to analysing the data and have revised the manuscript. HL has contributed in collecting the data and revising the manuscript. MK has been a general supervisor and has involved in revising the manuscript and has given the final approval of this article to be considered for publication. All authors read and approved the final manuscript.
